# Accurate expectancies diminish perceptual distraction during visual search

**DOI:** 10.3389/fnhum.2014.00334

**Published:** 2014-05-27

**Authors:** Jocelyn L. Sy, Scott A. Guerin, Anna Stegman, Barry Giesbrecht

**Affiliations:** ^1^Department of Psychological Sciences, Vanderbilt UniversityNashville, TN, USA; ^2^Department of Psychology, Yale UniversityNew Haven, CT, USA; ^3^Department of Psychological and Brain Sciences, University of CaliforniaSanta Barbara, Santa Barbara, CA, USA; ^4^Institute for Collaborative Biotechnologies, University of CaliforniaSanta Barbara, Santa Barbara, CA, USA

**Keywords:** selective attention, distraction, dorsal attention network, visual cortex

## Abstract

The load theory of visual attention proposes that efficient selective perceptual processing of task-relevant information during search is determined automatically by the perceptual demands of the display. If the perceptual demands required to process task-relevant information are not enough to consume all available capacity, then the remaining capacity automatically and exhaustively “spills-over” to task-irrelevant information. The spill-over of perceptual processing capacity increases the likelihood that task-irrelevant information will impair performance. In two visual search experiments, we tested the automaticity of the allocation of perceptual processing resources by measuring the extent to which the processing of task-irrelevant distracting stimuli was modulated by both perceptual load and top-down expectations using behavior, functional magnetic resonance imaging, and electrophysiology. Expectations were generated using a trial-by-trial cue that provided information about the likely load of the upcoming visual search task. When the cues were valid, behavioral interference was eliminated and the influence of load on frontoparietal and visual cortical responses was attenuated relative to when the cues were invalid. In conditions in which task-irrelevant information interfered with performance and modulated visual activity, individual differences in mean blood oxygenation level dependent responses measured from the left intraparietal sulcus were negatively correlated with individual differences in the severity of distraction. These results are consistent with the interpretation that a top-down biasing mechanism interacts with perceptual load to support filtering of task-irrelevant information.

## INTRODUCTION

Coping with the vast amount of complex information that is present in the environment requires the ability to selectively attend to information that is relevant for one’s behavioral goals while ignoring information that is irrelevant (Mangun and Buck, 1998; Chun and Marois, 2002). While selective processing is typically successful, attention can fail. These failures can result in interference or distraction from competing task-irrelevant features and objects (e.g., Stroop, 1935; Eriksen and Eriksen, 1974). Counterintuitively, increasing task demands at perceptual stages of visual processing can prevent these failures of selective attention (e.g., Norman and Bobrow, 1975; Yantis and Johnston, 1990; Lavie and Tsal, 1994; Lavie, 1995). For instance, in visual search tasks, increasing the number of items or the visual similarity between the target and distractors can reduce the behavioral interference caused by task-irrelevant objects (Lavie, 1995; Lavie and Cox, 1997). Similarly, decreasing the discriminability of task-relevant information can reduce the magnitude of neural responses evoked by irrelevant stimuli (Rees et al., 1997; Handy et al., 2001; Yi et al., 2004; Rorden et al., 2008).

The load theory of attention is one prominent explanation of how increased perceptual processing demands can reduce distractor interference (e.g., Lavie and Tsal, 1994; Lavie, 1995; Lavie et al., 2004). According to load theory, perceptual distraction occurs because at early stages of processing, limited resources are first allocated to task-relevant information and then any unused resources exhaustively and automatically “spill-over” to process irrelevant information. Distraction is reduced when perceptual demand, or load, is increased because fewer resources are available to “spill-over.” Critically, this explanation rests on two critical assumptions. First, the task-relevant and task-irrelevant information must be distinguishable in some fashion (e.g., by spatial location, color, size). Physical distinctiveness is important because it allows for top-down priorities to be set for allocating processing capacity to task-relevant information. Second, and most important for the present work, if relevant and irrelevant information are physically distinguishable, then the allocation of processing capacity to task-irrelevant information is determined automatically by the inherent perceptual demands imposed by the task-relevant information (Lavie, 1995, 2005; Lavie and Torralbo, 2010).

Although there is ubiquitous evidence demonstrating that task demands modulate distractor processing (Norman and Bobrow, 1975; Kahneman and Chajczyk, 1983; Dark et al., 1985; Lavie and Tsal, 1994; de Fockert et al., 2001; Handy et al., 2001; Lavie, 2005, 2010; Fu et al., 2010), several studies have provided evidence that distraction is not determined solely by the perceptual demands of the search display (Paquet and Craig, 1997; Johnson et al., 2002; Theeuwes et al., 2004; Biggs and Gibson, 2010; Chen and Cave, 2013; Benoni et al., 2014). For example, predictive spatial cues (Johnson et al., 2002) can eliminate behavioral distraction under low load conditions. Similarly, prior knowledge of the color of a target can influence both target and distractor processing (Chen and Cave, 2013). These studies are more in line with evidence that top-down expectancies can influence responses measured in visual cortex that represent both task-relevant (Kastner et al., 1998; Hopfinger et al., 2000; Giesbrecht et al., 2006) and task-irrelevant information (Serences et al., 2004). Interestingly, the blocked designs used in many load theory experiments suggest distractor interference can occur even when an accurate expectancy could be generated (e.g., Lavie, 1995). The difference between studies showing the influence of expectancies on selectivity and the typical load theory experiment may be that top-down expectations are used differently in experiments in which they can be generated on a trial-by-trial basis and those in which they can be generated for an entire block of trials (i.e., as in blocked-designs). Consistent with this interpretation, load effects can differ between blocked and intermixed designs, and trial-to-trial dependencies of load within intermixed experimental designs differentially modulate the magnitude of interference from irrelevant stimuli (Theeuwes et al., 2004; Biggs and Gibson, 2010; Benoni et al., 2014). For example, Theeuwes et al. (2004) compared perceptual load effects on distractor interference when load trials were blocked and intermixed. They found that the blocked design replicated previous demonstrations that distractor interference was larger under low load compared to high load. In contrast, the influence of perceptual load on flanker interference was not only less reliable in the intermixed trial design, but flanker interference was also modulated by the immediate trial history, suggesting that implicit task sets can impact flanker processing.

Given the discrepancy between the strong predictions of load theory and the behavioral evidence showing that perceptual distraction is not only driven by the load of the search display (e.g., Johnson et al., 2002; Theeuwes et al., 2004) and the neural evidence of top-down influences on visual responses (e.g., Kastner et al., 1998; Hopfinger et al., 2000; Corbetta and Shulman, 2002; Bressler et al., 2008; Szczepanski et al., 2010), the current work aimed to investigate the roles of perceptual load and top-down expectancies in perceptual distraction and the underlying neural mechanisms within an intermixed trial design. Specifically, the present study was designed to test the load theory assumption that perceptual processing capacity is allocated to irrelevant stimuli in an automatic and exhaustive fashion when capacity is not completely consumed by task-relevant information. In two experiments, observers performed a modified visual search task in which the perceptual load of the search display was manipulated and a task-irrelevant flanker was presented at a location where a target was never presented (e.g., Lavie and Cox, 1997). In other words, the task-relevant and task-irrelevant stimuli were distinguished based on physical location and the distinctiveness was constant across all conditions. The behavioral interference caused by the irrelevant flanker was used as a behavioral measure of perceptual distraction (e.g., Lavie, 1995). Critically, top-down expectancies were manipulated on each trial by presenting an explicit cue prior to the main search display indicating the most likely load of the task. Experiment 1 (Exp. 1) was a functional magnetic resonance imaging (fMRI) experiment in which we investigated the extent to which responses in visual cortex were modulated by cue-generated expectations and by the interaction between cue validity and search difficulty. Moreover, we also measured the effect of cue validity and search difficulty on blood oxygenation level dependent (BOLD) responses in the dorsal attention network because it is generally considered to mediate expectation-induced modulations of visual cortical responses (e.g., Kastner et al., 1998; Hopfinger et al., 2000; Corbetta and Shulman, 2002; Bressler et al., 2008; Szczepanski et al., 2010). Experiment 2 (Exp. 2) was an event-related potential (ERP) experiment that investigated the extent to which sensory processing of the flanker was modulated by cue-generated expectations and search difficulty within the first 200 ms of stimulus presentation. In both experiments we observed evidence for a reduction of the behavioral distraction effect when cues were valid, even under low task demands. Similarly, the neural evidence indicated more visuocortical selectivity in favor of relevant stimuli over the task irrelevant flanker and that the dorsal attention network was less affected by load when the cues were valid compared to when the cues were invalid. Together this evidence supports the notion that perceptual load was not the sole determinant of interference, but rather it is top-down expectations combined with the perceptual task demands that determine the magnitude of perceptual distraction.

## MATERIALS AND METHODS

### EXPERIMENT 1

#### Experiment 1A: predictive cue visual search task

***Participants***. Fourteen volunteers from the University of California Santa Barbara community were paid $20/h for their participation in the fMRI experiment (mean age = 24.8; seven female). All procedures conformed to a protocol approved by the University of California Santa Barbara Human Subjects Committee.

***Apparatus and Stimuli***. Stimulus presentation was controlled using custom scripts written in MATLAB (Mathworks, Inc., Boston, MA, USA) using the Psychophysics Toolbox (Brainard, 1997). Each search display consisted of a fixation circle (0.52°) presented at the center of each display. The search items were black upper case letters (Arial font, 0.52° tall) presented in the upper visual field on an arch 1.25° from fixation, and 0.95° from each other. The irrelevant flanker was also a letter (Arial font, 0.65° tall) and it was presented in the upper left or upper right of the display, 2.21° away from fixation (equidistant from the horizontal and vertical meridian).

**FIGURE 1:**
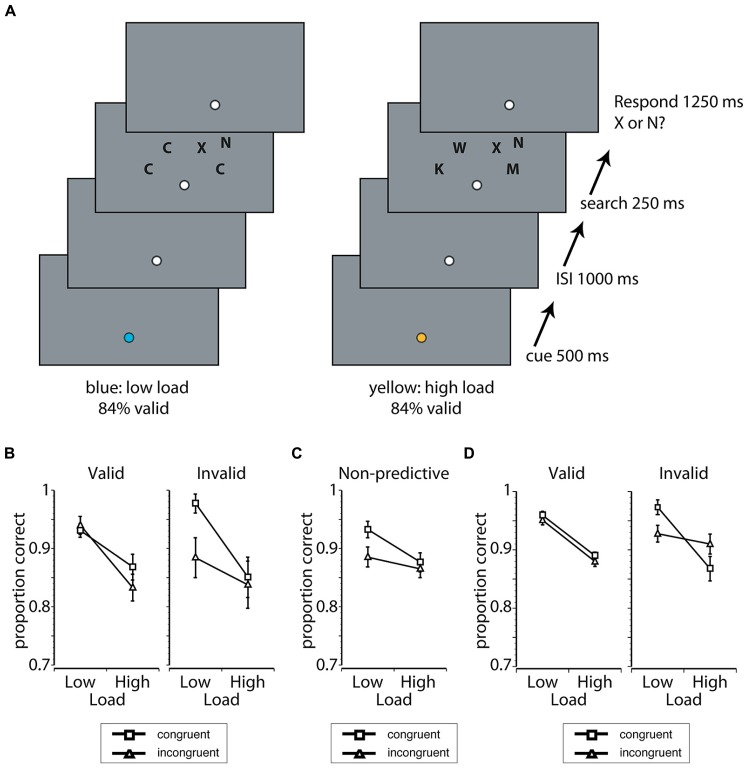
**(A)** A schematic of a cue + target trial sequence in Experiment 1 when participants were cued validly to a low load (left) or a high load (right) display by the color of fixation (blue or yellow). The load of the search display was manipulated while participants were instructed to discriminate whether an “N” or “X” was presented in the arch above fixation. On low load trials, the identities of the search distractors were randomly selected from a set of letters that had features dissimilar to the potential targets and all distractors on a given trial had the same identity. On high load trials, search distractors were randomly selected from a set of letters that had features similar to the potential targets and all distractors on a given trial had different identities. Behavioral interference and sensory processing of a flanker presented in an irrelevant search location, which was congruent or incongruent to the target, was used to index distraction. **(B)** Proportion of correct responses as a function of cue validity, load, and target-flanker congruency in Experiment 1A. Congruent trials are marked by squares, and incongruent trials are marked by triangles in all panels in this figure. Error bars represent the standard error of the mean (SEM). **(C)** Proportion of correct responses in Experiment 1B, the non-predictive control group. Error bars represent the SEM. **(D)** Proportion of correct responses in Experiment 2. Error bars represent the SEM.

***Procedure***. Each trial began when the fixation circle changed color for 500 ms. The specific color (blue or yellow) predicted the difficulty of the upcoming search display (see Design). After the cue, there was either a 250- or a 1000-ms delay during which the fixation point was white and remained on the screen, followed by the presentation of the search display (250 ms). Participants were instructed to discriminate whether an N or X was presented in one of four relevant locations in the arch above fixation and to ignore the irrelevant flanker presented either to the upper left or right of the search array. Participants were given a 1250 ms response interval to report the target. The instructions to the participants emphasized that it was important to make active use of the information provided by the cue on each trial, that it was important to be as accurate as possible, and that it was also important to maintain fixation throughout the trial. Once the trial was terminated by a response or the end of the response interval occurred, the fixation changed from white to an empty circle (a black outlined circle filled with the background color). A schematic of the trial sequence is shown in **Figure 1**.

***Design***. There were three critical manipulations. First, the perceptual load of the search task was manipulated in a manner consistent with previous manipulations that yielded results in favor of load theory (Lavie and Cox, 1997; Lavie, 2005; Roper et al., 2013). In the low load displays, the identities of the search distractors (i.e., items in potential target locations) were randomly selected from a set of letters that were featurally dissimilar to the potential targets (C, O, G, or Q). All the distractors on a given low load trial had the same identity (e.g., all O’s; Lavie and Cox, 1997). In the high load displays, the identities of the search distractors were randomly selected from a set of letters that were featurally similar to the potential targets (F, H, J, K, M, P, S, U, and W). The distractors on a given high load trial each had a unique identity (e.g., Lavie and Cox, 1997). Second, the cue (color change at fixation) predicted the upcoming load with 84% validity (16% invalid cue + target trials). The mapping of the cue color (blue or yellow) to the difficulty condition (low or high load) was counterbalanced across subjects. Third, a to-be-ignored flanker letter was presented unilaterally, either in the upper-left or upper-right of the search array and it was either congruent with the presented target (e.g., “X” target, “X” flanker) or incongruent (e.g., “X” target, “N” flanker). All factors were randomly intermixed within each run.

In addition to the cue + target trials described above, there were also trials in which the cue was presented, but no search array was presented (cue-only; 16% of trials). On the cue-only trials, the cue changed color as in the cue + target trials (e.g., blue for 500 ms, white for 1000 ms, and then empty until the next trial). The cue-only trials were included to estimate the BOLD response to the cues separately from the targets. There were also trials in which there were no stimulus events, cue or search array (null-event; 25% of trials). The null-event trials were included to facilitate the event-related analysis. Each participant performed seven fMRI scan runs, which included 96 total trials each (56 cue + target, 16 cue-only, and 24 null-event trials). Cue + target, cue-only, and null-event trials were the same duration and randomly intermixed within each run. Each experimental run started and ended with 12 s of blank fixation.

***Behavioral performance analysis***. Accuracy was chosen as the dependent measure to maximize the experiment’s sensitivity to changes in perceptual processing and to minimize speed-accuracy tradeoffs and motor biases that can occur when using reaction time as the dependent measure (e.g., Santee and Egeth, 1982; Prinzmetal et al., 2005; Bisley and Goldberg, 2006). Accuracy measures have successfully demonstrated perceptual load effects in distractor processing (Cartwright-Finch and Lavie, 2007; Konstantinou and Lavie, 2013). Behavioral interference scores were established for each participant as a function of cue validity and difficulty by calculating the difference in accuracy between congruent and incongruent trials.

***fMRI acquisition***. All fMRI data were collected using a 3-T Siemens Trio MRI scanner equipped with a standard 12-channel phased-array head coil located in the University of California Santa Barbara Brain Imaging Center. Whole-brain functional images were acquired using a T2^*^-weighted gradient-echo, echo-planar imaging sequence with a repetition time (TR) of 1.5 s, an echo time (TE) = 30 ms, and a 90° flip angle (FA). Each functional volume consisted of 28 interleaved slices acquired parallel to the anterior commissure–posterior commissure (AC–PC) line, voxel size of 3 mm × 3 mm × 4 mm, and field of view (FOV) of 192 mm × 192 mm. Anatomical images were acquired using a T1-weighted, spoiled gradient-echo sequence (MPRAGE; TR = 15 ms, TE = 4.2 ms, FA = 20°, voxel size of 0.9 mm × 0.9 mm × 0.9 mm, FOV = 240 mm × 240 mm).

***fMRI analysis***. Standard spatial preprocessing was applied using SPM5 (http://www.fil.ion.ucl.ac.uk/spm/). Images were realigned to the first functional image to correct for minor head motion. The mean functional image was coregistered to the anatomical image and all functional images were coregistered to the mean functional image. The functional and anatomical images were normalized to conform to the MNI-152 template. The normalized functional images were then spatially smoothed with a 6-mm^3^ isotropic Gaussian kernel. SPM5 was used to implement a voxel-wise least-squares general linear model that did not assume an *a priori* shape for the hemodynamic response (Ollinger et al., 2001a,b). Regressors were included for eleven peristimulus time-points associated with the cue onset. A unique set of 11 temporal parameters was modeled for high and low load valid cue, high and low load invalid cue, high and low load cue-only, and null-event trials irrespective of stimulus onset asynchrony (SOA). The data were concatenated across the seven functional runs and stimulus effects were modeled by the same parameters across all runs; the model did not assume temporal continuity between runs. To correct for linear drift and mean run effects, a constant and a linear drift term were included separately for each run. All whole-brain contrasts compared model-estimates at the peak time-point, estimated to be 3–7.5 s after stimulus presentation.

Regions of interest (ROIs) were identified as those clusters that survived the cue-only trials versus null-event contrast, thresholded at *p* < 0.05 false-discovery rate (FDR) corrected with 10 contiguous voxels. Due to a response recording error, all subsequent behavioral and neuroimaging analyses of the Exp. 1 and Exp. 2 data focused on the longer (1500 ms) cue + target SOA condition. Within these cue-only ROIs, the average event-related hemodynamic responses evoked by the cue + target and null-event trials were calculated. Average event-related hemodynamic responses were converted to percent signal change relative to a baseline that included the averaged signal intensity at the onset of the cue display and the immediately preceding time-point. Overlap in the hemodynamic responses to the search trials in this fast-rate design was corrected by subtracting the hemodynamic responses corresponding to the null-event trials (Burock et al., 1998; Woldorff et al., 2004; Giesbrecht et al., 2013). The resulting signal change data on cue + target trials were entered into separate repeated-measures ANOVAs for each region, using cue validity, load, and sampled time point as factors.

Individual differences in peak percent signal change on both valid and invalid low load cue + target trials within the ROIs identified by the cue-only contrast were also correlated with individual differences in behavioral interference scores. Correlation analyses were restricted to the low load conditions (valid and invalid cues) given that these were the conditions predicted, *a priori*, to have the greatest amount of interference.

***Effect sizes***. Effect sizes for all hypothesis tests conducted in each experiment were computed. Specifically, for hypothesis tests conducted using ANOVA, $\eta_{partial}^{2}$ is reported. For hypothesis tests using the *t*-statistic, Cohen’s *d* is reported. Small, medium, and large effects correspond to $\eta_{partial}^{2}$ values of approximately 0.01, 0.06, and 0.14 and correspond to Cohen’s *d* values of approximately 0.2, 0.5, and 0.8, respectively (e.g., Cohen, 1992).

#### Spatial localizer task

***Participants***. The same 14 volunteer participants in the cued search fMRI task were exposed to separate spatial localizer tasks in the same experimental session. One subject was excluded from all visual cortical analyses because portions of visual cortex were cut off during fMRI scans. Two other participants were excluded from the group localizer whole brain contrasts, due to missing event onsets for spatial localizer runs. However, these two participants were included in the selective averaging analysis using ROIs established by the group localizer contrasts.

***Apparatus and stimuli***. Each display consisted of a fixation cross in the center of screen and a flickering black and white checkered circle (0.52° in diameter) presented in each hemifield. The checkered circles were placed in spatial locations corresponding to the relevant search or irrelevant flanker locations in the cued search task. Black and white circles flickered in each relevant and irrelevant spatial location independently at 15 Hz. The color of fixation changed from white to red for 27 ms at random intervals.

***Procedure***. Participants were instructed to fixate on and press a button when they detected a color change of the centrally presented fixation cross. Independent of the color detection task, spatial locations associated with relevant search and irrelevant flanker locations in the cued search task were stimulated with flickering black and white circles in both hemifields in approximately 12 second blocks. The duration a spatial location was stimulated ranged from 12 to 13.5 s depending on the number of fixation color changes that occurred in the block. Flickering stimuli were first presented in the two lateral search locations, then two medial locations, and then two flanker locations, in four repetitions per localizer run. Participants performed two localizer runs, totaling eight stimulus repetitions for each spatial location.

***fMRI analysis***. All fMRI data for the spatial localizer task were preprocessed in the same fashion as the fMRI data collected during the search task. To correct for linear drift and mean session effects, a constant and a linear drift term were included separately for each session. A canonical hemodynamic response function was used to model activation for each stimulated spatial location. ROIs were identified as those clusters posterior in the brain that survived the weighted contrast between two bilateral relevant spatial locations versus one bilateral irrelevant spatial location (*p* < 0.005, uncorrected, 10 contiguous voxels). Within the surviving ROIs, the average event-related hemodynamic response recorded during the cue-only and cue + target trials in the search task was calculated and converted to percent signal change. The mean peak percent signal change for cue-only trials in each visual cortical ROI was entered into a repeated-measures ANOVA using ROI and predicted difficulty as factors. Additionally, the mean peak percent signal change on cue + target trials in each visual cortical ROI was entered into a repeated-measures ANOVA using ROI, cue validity, and difficulty as factors.

#### Experiment 1B: non-predictive cue visual search task

A different set of 36 volunteers (mean age = 19.9; 28 female) participated in a non-predictive cue search task that served as a behavioral control group for the predictive cue search task described above. The stimuli and timing were exactly the same as the predictive cue task, except that the color of the fixation circle was not predictive of the upcoming load (or other display parameters). The non-predictive control experiment was conducted in order to verify whether the task and display would sufficiently induce the typical interaction between perceptual difficulty and flanker interference (e.g., Lavie and Cox, 1997) when using accuracy as a dependent measure. Performance in the non-predictive group was used as a statistical baseline and was separately compared to validly and invalidly cued conditions in Exp. 1A. Difficulty and flanker-target congruency were used as within subjects factors and experimental group (predictive versus non-predictive cues) as a between subjects factor in a repeated-measures ANOVA for behavioral analysis.

### EXPERIMENT 2

#### Participants

Fifteen volunteers from the University of California Santa Barbara community were paid $10/h for their participation (mean age = 20; nine female). All procedures conformed to a protocol approved by the University of California Santa Barbara Human Subjects Committee. Two participants were excluded from analyses because they failed to demonstrate a measurable positive P1 component.

#### Apparatus, stimuli, procedure, and design

All aspects of this experiment were the same as Exp. 1, except that one item was added to the display. This change was made to match the mean level of behavioral performance in Exp. 1 because pilot testing revealed overall higher performance in the electroencephalography (EEG) chamber compared to the scanning environment. The difference in performance was likely due to the difference in display contrast and ambient lighting in the scanner and EEG recording environments. The resulting five search locations were evenly distributed in an arch (1.25°) above fixation, and 0.65° from each other. Participants were exposed to 11 experimental runs.

#### Behavioral performance analysis

Accuracy was analyzed as a function of cue validity, load, and flanker-target congruency using a repeated-measures ANOVA.

#### EEG acquisition and analysis

EEG activity was recorded inside an 8′ × 10′ electromagnetic field shielded chamber (ETS-Lindgren, Cedar Park, TX, USA). Data were sampled at 512 Hz using 32 Ag/AgCl sintered electrodes placed according the International 10/20 System on the scalp and referenced offline to the Cz electrode. Next, the EEG signal was band-pass filtered between 0.1 and 30 Hz to exclude low and high frequency noise. To ensure fixation, trials containing ocular artifacts, measured by the bipolar electrooculogram (EOG) amplitudes exceeding ±75 μV, were excluded from analysis. The mean percentage of rejected trials across subjects was 6%. The remaining EEG signals were averaged from 100 ms prior to and 400 ms after the onset of the search display to produce the ERP response.

The mean amplitude of the P1 ERP component was calculated as a function of cue validity and difficulty. The mean amplitude of the P1 was computed by first identifying the peak of the first positive deflection observed between 75 and 200 ms after the onset of the search display. This was done for each participant using the average waveform collapsed across all conditions over posterior occipital electrodes (PO3, O1, PO4, O2; Heinze et al., 1990; Heinze and Mangun, 1995; Wijers et al., 1997). On average, the mean latency of the P1 peak amplitude measured at electrodes contralateral to the irrelevant flanker was 130 ms after the onset of the search display. Finally, the mean amplitude for each condition was computed within a 45-ms time window centered on the identified peak. The mean P1 amplitude was entered into a repeated-measures ANOVA with cue validity and load as within subjects factors.

## RESULTS

### EXPERIMENT 1

We used the load-cue search task to investigate three possible effects that top-down expectations could have on perceptual distraction. First, if the processing of irrelevant information is automatically determined by perceptual demands, as predicted by load theory, then flanker interference should be larger under low load than high load conditions, regardless of the top-down expectancy engendered by the cue. Second, if the processing of irrelevant information is driven solely by top-down biasing signals, then the expectations induced by the cue should affect the amount of flanker interference and the neural responses evoked by the flanker. This top-down influence could take multiple forms. For example, if perceptual distraction is driven entirely by top-down expectations concerning the difficulty of the upcoming search display, then flanker interference could be greater when participants expect low load (i.e., valid low and invalid high demand trials) than when they expect high load (i.e., valid high and invalid low demand trials). In other words, perceptual distraction could depend on one’s top-down expectations regardless of whether those expectations turn out to be correct (i.e., on valid trials) or incorrect (i.e., on invalid trials). That said, previous work shows that behavioral distraction (Johnson et al., 2002) and baseline responses in regions of visual cortex that represent irrelevant spatial locations (Serences et al., 2004) are reduced by expectations despite low perceptual demands. Thus, another form of top-down influence is that cued-induced expectancies will reduce or eliminate sensory processing of the flanker and the corresponding behavioral interference when expectations are accurate (i.e., valid) but not when they are inaccurate (i.e., invalid), *and* this effect will occur regardless of the difficulty of the search task. In other words, when the cues are valid, there will be no flanker interference in either the low or the high load conditions, but when the cues are invalid, there could be more flanker interference compared to valid trials. Third, the allocation of resources could be determined by the interaction between top-down and more bottom-up perceptual processing mechanisms. If true, then cue-evoked expectations may interact with the difficulty of the search display to modulate both behavioral and neural measures of distraction. For example, based on the work cited above (e.g., Johnson et al., 2002; Serences et al., 2004), it could be the case that accurate (i.e., valid) top-down expectations concerning search difficulty may serve to eliminate distraction regardless of the perceptual load of the display; in contrast, inaccurate (i.e., invalid) top-down expectations may allow distraction to be more strongly influenced by the perceptual load of the display.

Importantly, if load- and expectation-mediated effects on behavioral distraction reflect early sensory processing mechanisms, visual cortical responses measured with fMRI should mirror the behavioral modulations of distraction similar to one of the three possible effects outlined in the preceding paragraphs. In addition, we predicted that evidence for top-down influences on perceptual distraction would also be reflected at the level of large-scale cortical networks, including the frontal and parietal regions associated with the volitional control of attention (Kastner et al., 1998; Hopfinger et al., 2000; Corbetta and Shulman, 2002; Yantis et al., 2002; Giesbrecht et al., 2003; Kincade et al., 2005).

#### Behavioral performance

***Expectation-induced modulations of behavioral distraction***. Behavioral distraction was indexed by the difference in accuracy when the irrelevant flanker was congruent with the target versus when the flanker was incongruent. Behavioral performance in the predictive cue search task (Exp. 1A) is shown in **Figure 1B** alongside the results of the non-predictive control condition (Exp. 1B) in **Figure 1C**. When cues accurately predicted load (e.g., low load cue, low load display) there was a significant interaction between load, flanker congruency, and experimental group [*F*(1,48) = 5.29, *p* = 0.026,$\eta_{partial}^{2}$ = 0.10]. There were no other main effects or interactions with experimental group (all *p*’s > 0.06, $\eta_{partial}^{2}$ < 0.08). In order to understand the nature of the significant three-way interaction, *post hoc* analyses were conducted for the valid cue condition (Exp. 1A) and the non-predictive group (Exp. 1B) separately using difficulty and flanker congruency as factors. In the non-predictive group, there was a significant difficulty × congruency interaction, such that flanker interference was larger with low load displays than with high load displays [*F*(1,35) = 4.43, *p* = 0.042, $\eta_{partial}^{2}$ = 0.11]. The observed interaction in the non-predictive group confirmed that the use of accuracy as a dependent measure successfully induced the pattern of behavior predicted by the load theory of attention, in the absence of top-down expectancy. In contrast to the non-predictive group, congruency had no main effect and did not interact with difficulty in the valid cue condition (all *p*’s > 0.16, $\eta_{partial}^{2}$ < 0.14). The absence of a congruency effect indicated that distraction in both difficulty conditions was eliminated when difficulty expectations were accurate.

The analysis comparing the invalid cue condition to the non-predictive group revealed a significant interaction between flanker congruency and difficulty [*F*(1,48) = 6.75, *p* = 0.012, $\eta_{partial}^{2}$ = 0.12]. This interaction was driven by more flanker interference under low load compared to high load. No other main effects or interactions with experimental group were significant (all *p*’s > 0.17, $\eta_{partial}^{2}$ < 0.04). Critically, this analysis demonstrated that distraction was driven by the difficulty induced by the search display when expectations were incorrect (Exp. 1A) or absent (Exp. 1B).

#### Functional magnetic resonance imaging

The fMRI data were analyzed to address two key issues. The first was to determine whether the interaction between perceptual load and top-down expectation was present in BOLD responses in visual cortex. Second, we investigated whether regions of the dorsal attention network, typically associated with voluntary control (Kastner et al., 1998; Hopfinger et al., 2000; Corbetta and Shulman, 2002; Kincade et al., 2005), play a role in mediating distraction.

**FIGURE 2:**
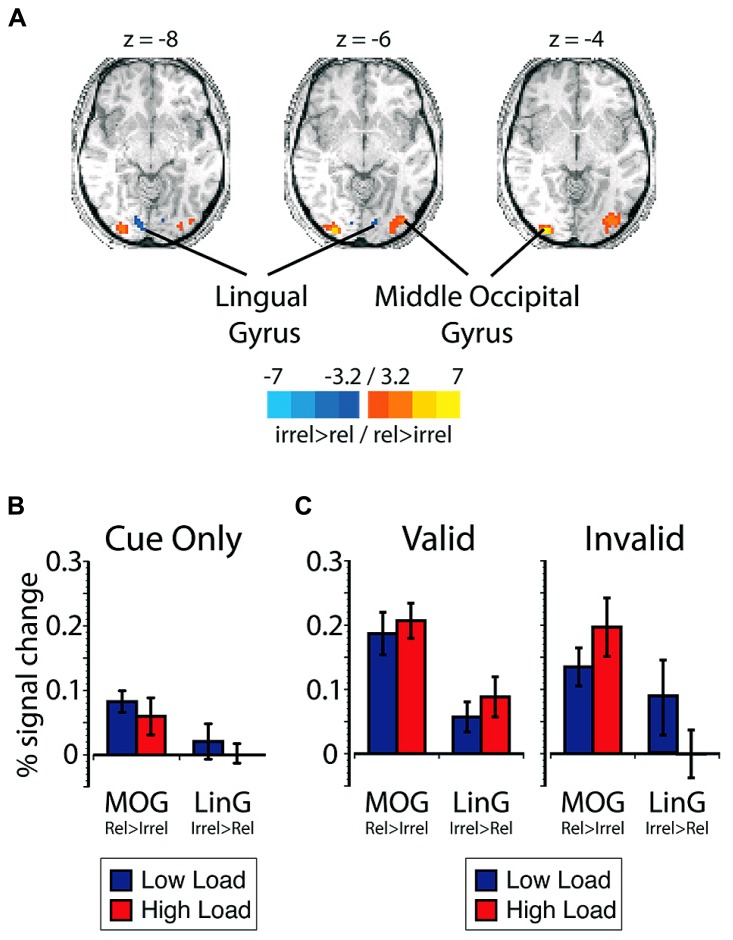
**(A)** Data from the spatial localizer task showing regions with greater selectivity to relevant search locations compared to irrelevant flanker locations. Scale corresponds to the *t*-value. The left side of the brain is depicted on the left side of the figure. The peak percent signal change is plotted in blue for low load and red for high load **(B)** on cue-only trials within visual cortex ROIs plotted as a function of ROI, and **(C)** on cue + target trials within visual cortex ROIs plotted as a function of cue validity and ROI. Error bars plotted within this figure represents the standard error of the mean appropriate for within-subjects comparisons (Loftus and Masson, 1994).

***Expectation-induced modulations in visual cortex***. First, to investigate the effect of expectations on visual sensory processing in our task, regions of visual cortex that represented the relevant search and the irrelevant flanker locations were identified using an independent spatial localizer (see Materials and Methods). The region that responded more robustly to stimuli presented in the relevant search locations than to stimuli presented in the irrelevant flanker locations included the middle occipital gyrus (MOG_rel > irrel_; **Figure 2A**, **Table 1**). The region that preferentially responded to stimuli presented in irrelevant flanker locations compared to relevant search locations included the lingual gyrus (LinG_irrel > rel_). Given the predicted tradeoffs between relevant and irrelevant information processing (Lavie and Tsal, 1994; Lavie, 1995; Lavie et al., 2004), perceptual selectivity in favor of relevant information was indexed in the main experimental runs by larger mean BOLD responses in MOG_rel > irrel_ than LinG_irrel > rel_.

**Table 1:** Regions of significant activation in the whole-brain contrast for the spatial localizer task.

Region	*x*	*y*	*z*	Voxels
**Relevant > irrelevant**
Left middle occipital gyrus	-30	-96	-3	82
Right middle occipital gyrus	36	-78	-0	82
Left precuneus	0	-51	57	23
Left superior frontal gyrus	-12	12	57	10
**Irrelevant > relevant**
Right anterior cingulate	15	18	24	14
Left lingual gyrus	-12	-87	-9	25
Right lingual gyrus	12	-87	-6	12

*MNI coordinates represent local *t*-maximum for clusters thresholded at *p* < 0.005 uncorrected, with 10 voxels spatial extent.*

To determine if expectations influenced preparatory responses, BOLD responses on cue-only trials in regions of visual cortex that represented the search and flanker locations were analyzed as a function of ROI (i.e., MOG_rel > irrel_ and LinG_irrel > rel_) and cued load. There were no significant main effects or interactions with either ROI or cued load (*p* > 0.08; $\eta_{partial}^{2}$ < 0.23; **Figure 2B**).

To investigate the extent to which search load and expectation influenced visual sensory processing during visual search, visual cortical responses on cue + target trials were compared as a function of cue validity and load. There was a significant three-way interaction between ROI, load, and cue validity [*F*(1,12) = 14.64, *p* = 0.002, $\eta_{partial}^{2}$ = 0.55; **Figure 2C**]. Separate *post hoc* repeated-measures ANOVAs revealed that the three-way interaction was driven by the fact that there was an interaction between ROI and search load on invalidly cued trials [*F*(1,12) = 11.36, *p* = 0.006, $\eta_{partial}^{2}$ = 0.49]. This interaction was due to a larger BOLD response in MOG_rel > irrel_ than in LinG_irrel > rel_ on high load trials [*t*(12) = 2.98, *p* = 0.012, *d* = 0.84]. The larger response in MOG_rel > irrel_ suggests perceptual selectivity in favor of relevant search locations over irrelevant flanker locations. On low load trials, however, the response in MOG_rel > irrel_ was not different from LinG_irrel > rel_ [*t*(12) = 0.83, *p* = 0.42, *d* = 0.23]. The similar response magnitudes in MOG_rel > irrel_ and LinG_irrel > rel_ suggest that there was relatively comparable levels of visual processing in these locations and weak perceptual selectivity. The pattern of activation on invalid trials is consistent with predictions of load theory and previous demonstrations that perceptual load modulate visual responses (Rees et al., 1997; Handy et al., 2001; Yi et al., 2004; Rorden et al., 2008). In contrast to invalid trials, MOG_rel > irrel_ exhibited larger responses than LinG_irrel > rel_ on validly cued trials [*F*(1,12) = 6.40, *p* = 0.026, $\eta_{partial}^{2}$ = 0.35], but the difference between ROIs did not interact with search load [*F*(1,12) = 0.40, *p* = 0.54, $\eta_{partial}^{2}$ = 0.03]. The larger response in MOG_rel > irrel_ than in LinG_irrel > rel_ supports the notion that relevant search locations were selectively enhanced compared to the irrelevant flanker locations when the cue was valid, regardless of perceptual load. As a whole, the three-way interaction between ROI, difficulty, and cue validity is consistent with the notion that top-down expectation and perceptual load interact in visual cortex to influence the extent to which relevant and irrelevant information is processed.

**FIGURE 3:**
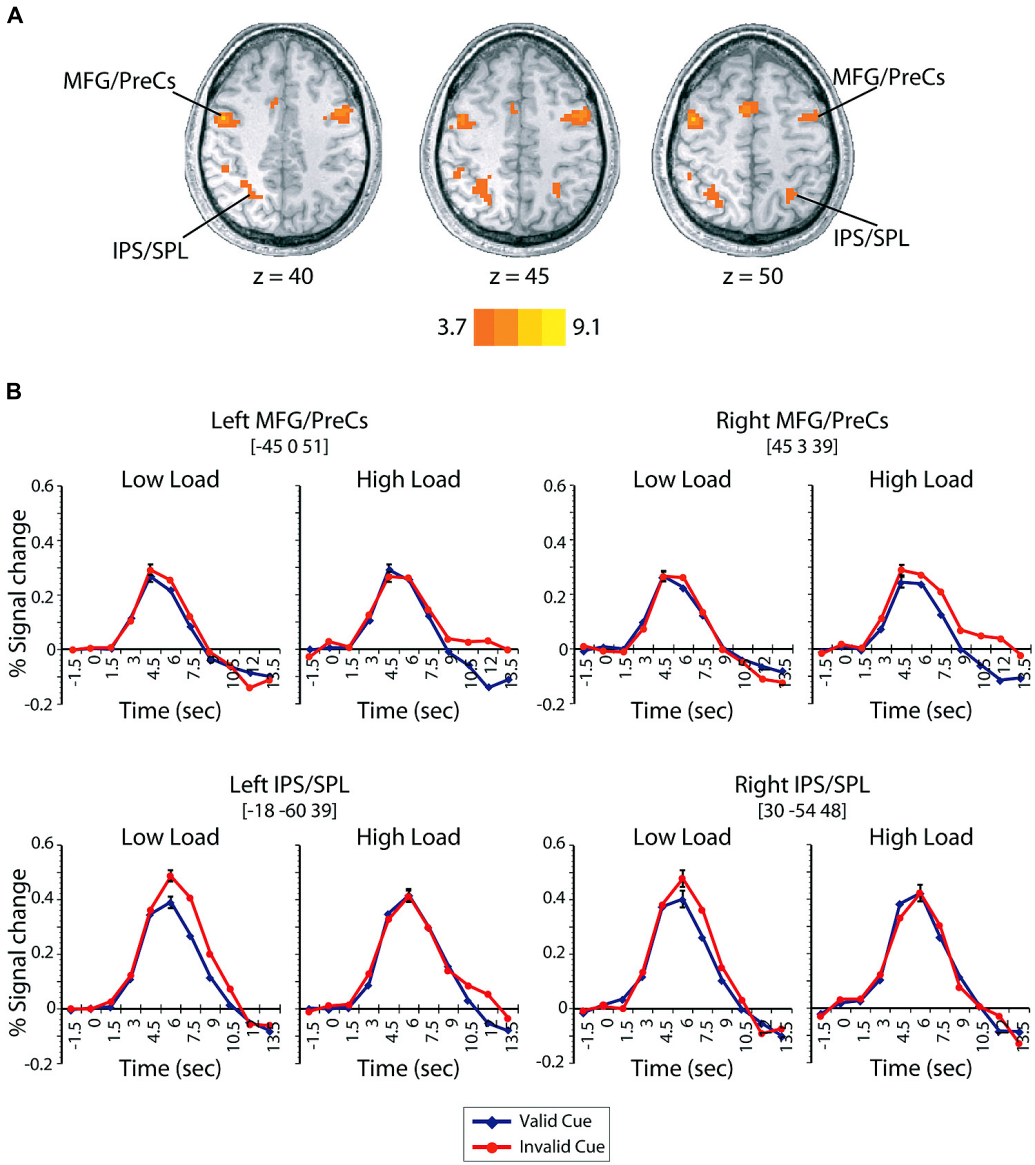
**(A)** Regions demonstrating greater activations in cue-only trials compared to null-event baseline. The left side of the brain is depicted on the left side of the figure. Scale corresponds to the *t*-value. **(B)** Time-course of BOLD signals on valid (blue) and invalid (red) cue + target trials as a function of load and time in the dorsal frontoparietal control network (bilateral MFG/PreCs and bilateral IPS/SPL). Error bars represent the mean squared error of the interaction between cue validity, load, and time.

***Expectation induced modulations in the dorsal attention network***. To investigate the role played by the dorsal attention network in mediating distraction, we first identified regions that were engaged on trials that contained a cue but no search display (Woldorff et al., 2004; Slagter et al., 2006). This contrast isolated regions of activation within the dorsal frontoparietal voluntary attention network – bilateral dorsal parietal areas, including the intraparietal sulcus (IPS) extending into the superior parietal lobe (SPL), and bilateral dorsal lateral prefrontal cortex, including the posterior aspect of the middle frontal gyrus (MFG) extending into the precentral sulcus (PreCs; **Figure 3A**; **Table 2**).

**Table 2:** Whole-brain contrast for cue-only minus null-event activation.

Region	*x*	*y*	*z*	Voxels
Left middle occipital gyrus	-30	-90	-3	194
Right middle frontal gyrus/precentral sulcus	45	3	39	162
Right occipital/fusiform	36	-66	-15	112
Left middle frontal gyrus/precentral sulcus	-45	0	51	159
Left superior frontal gyrus	0	9	57	128
Right insula	36	21	12	37
Left posterior cingulate	0	-30	21	38
Right superior temporal gyrus	48	-36	15	70
Left intraparietal sulcus/superior parietal lobe	-18	-60	39	120
Left superior temporal gyrus	-51	-42	21	23
Right intraparietal sulcus/precuneus	30	-54	48	37

*MNI coordinates represent local *t*-maximum for clusters thresholded at *p* < 0.05 FDR corrected, with 10 contiguous voxels.*

To assess the involvement of these regions in mediating distraction during visual search, we extracted the percent signal change on cue + target trials within each ROI of the dorsal frontoparietal network identified by the cue-only contrast. If the dorsal frontoparietal network is involved in mediating distraction, regions within this network should exhibit interactions between cue-validity and load similar to those observed in the patterns of behavioral performance and BOLD responses in visual cortex. The resulting BOLD time-courses are shown in **Figure 3B**. Activity on cue + target trials within the bilateral dorsal parietal and frontal regions associated with voluntary control revealed two key findings. First, the left parietal region, IPS/SPL, demonstrated a significant interaction between cue validity, load, and time [*F*(10,130) = 3.02, *p* = 0.002, $\eta_{partial}^{2}$ = 0.19]. Separate *post hoc* repeated-measures ANOVAs revealed that the three-way interaction was driven by the fact that the BOLD response in this region was not influenced by load on validly cued trials (*p* > 0.19, $\eta_{partial}^{2}$ < 0.10), but was modulated by the interaction between load and time on invalid trials [*F*(10,130) = 2.34, *p* = 0.014, $\eta_{partial}^{2}$ = 0.15]. Additionally, separate *post hoc* repeated-measures ANOVAs analyzing the difficulty conditions separately revealed no influence of cue validity on high load trials (*p* > 0.14, $\eta_{partial}^{2}$ < 0.10), but did reveal that the interaction between cue validity and time modulated the BOLD response in low load conditions [*F*(10,130) = 2.70, *p* = 0.005, $\eta_{partial}^{2}$ = 0.17]. There was an overall larger response on invalidly cued low load trials compared to validly cued low load trials. The pattern of activation in this region paralleled the pattern found in behavior, such that the invalid low load condition produced both the largest BOLD response and the largest behavioral distraction effect. The right parietal ROI, including IPS/SPL, exhibited a qualitatively similar response, but the three-way interaction was not significant [*F*(10,130) = 0.858, *p* < 0.57, $\eta_{partial}^{2}$ = 0.06]. The consistent interaction between cue validity and load observed in both behavior and BOLD responses in the left dorsal parietal region suggests that this region – which has been previously associated with directing spatial attention (Posner et al., 1984; Hopfinger et al., 2000; Corbetta and Shulman, 2002; Yantis et al., 2002; Woldorff et al., 2004), distractor inhibition (Friedman-Hill et al., 2003; Mevorach et al., 2006; Akyürek et al., 2010; Melloni et al., 2012), calculating perceptual saliency (Geng and Mangun, 2009), and working memory load (Todd and Marois, 2004) – is unaffected by perceptual difficulty when cues are valid but is affected by perceptual difficulty when cues are invalid.

The second key finding was that there were also significant interactions between cue validity, load, and time in both left [*F*(10,130) = 3.90, *p* < 0.001, $\eta_{partial}^{2}$ = 0.23] and right [*F*(10,130) = 2.70, *p* = 0.005, $\eta_{partial}^{2}$ = 0.18] dorsal lateral prefrontal ROIs (MFG/PreCs). The pattern of these interactions did not parallel the pattern of behavioral and perceptual distraction but did reveal that these regions had a larger response at later time points on invalidly cued high load trials compared to all other conditions. The interactions between cue validity, load, and time in dorsal frontal regions suggests that areas previously associated with global task difficulty (Demeter et al., 2011) and online switches of the task set in response to violations of expectancy (Sohn and Carlson, 2000; Koechlin et al., 2003; Sylvester et al., 2003) are engaged for a longer period of time on invalidly cued high load trials. While it is difficult to make strong inferences about the temporal dynamics of neural activity based on BOLD responses, the greater response at later time points on invalid high load trials may index differences in task difficulty and implicate greater demand on invalid high conditions compared to valid high, valid low, and invalid low load conditions. However, a *post hoc* comparison of behavioral accuracy between valid (*M* = 0.85) and invalid high (*M* = 0.84) load trials demonstrated no significant differences in overall accuracy [*t*(13) = 0.28, *p* = 0.78, *d* = 0.08] suggesting that the tasks were relatively equated in difficulty. Alternatively, the larger response in MFG/PreCs in later time points may also reflect a readjustment of the task set from an easy strategy consisting of searching for a salient feature to a more effortful search strategy requiring the conjunction of multiple features and the examination of several likely targets (Treisman and Gelade, 1980; Wolfe, 1994). It is possible that this later response is not present on invalid low load conditions because a salient target can be efficiently detected whilst maintaining the more difficult conjunction search strategy, thereby not requiring a switch in task set.

***Individual differences in distraction***. Parietal cortex, including both IPS and SPL, is functionally heterogeneous. For instance, dorsal parietal regions have previously been associated with the (1) orienting of attention toward relevant (Posner et al., 1984; Corbetta et al., 2000; Hopfinger et al., 2000; Corbetta and Shulman, 2002; Yantis et al., 2002; Woldorff et al., 2004) and away from irrelevant information (Friedman-Hill et al., 2003; Mevorach et al., 2006; Akyürek et al., 2010; Melloni et al., 2012); (2) efficient perceptual decision making (Kayser et al., 2010; Liu and Pleskac, 2011; Hebart et al., 2012); (3) integrating top-down and bottom-up saliency (Geng and Mangun, 2009); and (4) indexing the complexity or amount of information held within working memory (e.g., Todd and Marois, 2004). These proposed roles of parietal cortex make opposing predictions about the correlation between individual differences in behavioral distraction and BOLD responses within parietal cortex. Specifically, if the parietal cortex BOLD responses observed here are associated with successful attentional control or efficient perceptual decision making, then increases in the BOLD response should be associated with less perceptual distraction (i.e., a negative correlation). In contrast, if the parietal cortex BOLD responses observed here are indexing working memory load, then increases in the BOLD response should be associated with more perceptual distraction because the inclusion of the irrelevant flanker in working memory would also increase its potential for behavioral interference (i.e., a positive correlation).

**FIGURE 4:**
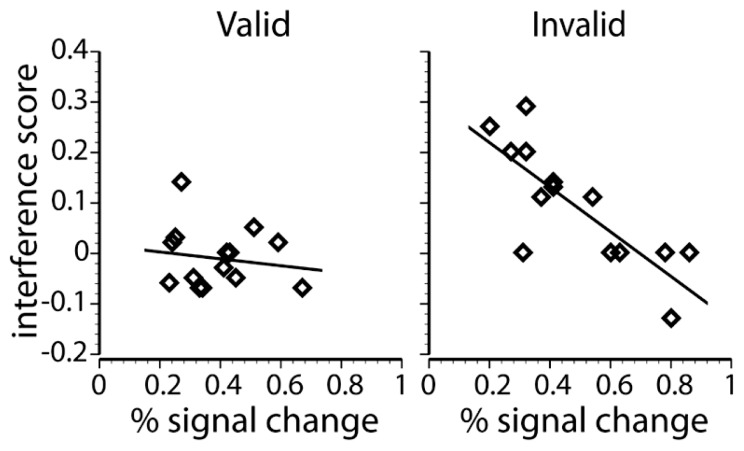
**Regression scatterplots that show the relationship between the peak percent signal change in the left IPS and the behavioral interference score for each individual subject on valid low load trials (left) and invalid low load trials (right)**.

To test these competing predictions, correlations between individual differences in behavioral distraction and responses in the voluntary control network were performed. The analyses correlated each individual’s peak percent signal change on cue + target trials within each of the four dorsal frontoparietal ROIs (identified by the cue-only contrast) with the size of each individual’s distraction effect observed in the valid and invalid low load conditions of Exp. 1A. Behavioral distraction was indexed by computing the performance (i.e., accuracy) difference between the congruent and incongruent flanker conditions. The correlation analyses were restricted to the low load conditions because this was the condition predicted to show behavioral distraction. All correlations were Bonferroni corrected (*p* < 0.05, two-tailed) for eight comparisons (4 ROI locations × 2 validity conditions), resulting in a threshold of *p* = 0.00625 for each test. The results of this correlation analyses are presented in **Table 3** and **Figure 4**.

**Table 3:** Pearson correlations between peak frontoparietal activations and behavioral interference scores under low load conditions.

Region	Valid cue	Invalid cue
Left precentral gyrus/middle frontal gyrus	0.25	-0.32
Right middle frontal gyrus	0.39	-0.20
Left precuneus/superior parietal lobe	-0.14	-0.81**
Right intraparietal lobule/precuneus	0.11	-0.55*

p < 0.05 two-tailed, uncorrected; **p < 0.01 two-tailed, Bonferroni corrected.

The percent signal change in the left parietal ROI and behavioral interference on invalidly cued low load trials had a strong negative correlation, *r*(12) = -0.81, *p* < 0.001, showing that individuals that exhibited relatively large BOLD responses in left IPS/SPL tended to exhibit smaller amounts of flanker interference. This correlation is consistent with the notion of a top-down biasing signal and efficient perceptual processing, suggesting that across individuals, increased involvement of dorsal partial cortex corresponds to reduced behavioral distraction. Interestingly, despite the identical display on validly cued low load trials, there was no correlation between BOLD responses and behavioral performance [*r*(12) = -0.14, *p* = 0.633]. Moreover, a direct comparison between the two correlation coefficients revealed that the correlation on invalid low load trials was different than the correlation on valid low load trials (z = 2.31, *p* = 0.01). There was also a trend for a similar relationship in the right parietal region, but it failed to reach significance when corrected for multiple comparisons [valid trials: *r*(12) = 0.11, *p* = 0.71; invalid trials: *r*(12) = -0.55, *p* = 0.04]. These results contrast previous findings by Kim and Hopfinger (2010) that the activation of the right dorsal parietal cortex positively correlated with attentional capture of novel objects and may reflect different sub-functions of this region (Corbetta and Shulman, 2002; Mevorach et al., 2006). The correlation between IPS/SPL BOLD responses and individual differences in distraction suggests that the interaction between validity and difficulty in this region is not due to more information being encoded into working memory. Rather, the results observed in IPS/SPL could represent more efficient filtering of task-irrelevant information or perceptual decision-making when faced with unexpected task demands.

### EXPERIMENT 2

Experiment 2 was an ERP experiment that used the same task to investigate the extent to which sensory processing of the flanker within the first 200 ms after the presentation of the search display was modulated by the interaction between cue-generated expectations and task demands. Exp. 2 focused on temporally early measures of sensory processing in order to establish if the effects of expectation and perceptual difficulty observed in Exp. 1 were representative of post-perceptual feed-back mechanisms involved in cognitive control (Lavie et al., 2004; Kelley and Lavie, 2011) or representative of early selection mechanisms associated with sensory processing. The difficulty of target processing has previously been shown to modulate the amplitude of the P1 ERP evoked by an irrelevant stimulus (Handy et al., 2001) and to a non-predictive peripheral spatial cue (Fu et al., 2010). Given the prior evidence supporting perceptual load modulations of the P1 and our primary interest in temporally early sensory processing, we measured the P1 ERP component at electrodes contralateral to the position of the irrelevant flanker. The P1 component is the first positive deflection in the ERP measured at lateral occipital scalp locations contralateral to the presentation of a visual stimulus and it is typically larger when the stimulus is attended relative to when it is unattended (Van Voorhis and Hillyard, 1977; Mangun and Hillyard, 1991; Heinze et al., 1994).

We hypothesized three possible effects of expectation and task load on the magnitude of the P1 associated to the irrelevant flanker. First, if the allocation of early visual processing resources depends on the perceptual load induced by task-relevant information alone, as predicted by load theory, then the amplitude of the P1 component measured at electrodes contralateral to the side of the irrelevant flanker should be modulated by load alone and should not be modulated by cue validity or expected load. As a result, any interactions between perceptual load and top-down expectation found in Exp. 1 would likely to represent top-down biases in later stages of cognitive control and selection from working memory (Lavie et al., 2004; Kelley and Lavie, 2011). Therefore, any influence of expectation should occur in ERP components associated with later cognitive stages of information processing. A second possible outcome is that expectations of difficulty solely influence early sensory processing and attentional selection. If top-down expectation dictates distraction, cue meaning (i.e., low load versus high load) or validity should modulate the amplitude of the P1. Finally, if early sensory processing is mediated by the combination of expectations and perceptual demands, then the amplitude of the P1 should be modulated by the interaction between cue validity and load.

#### Behavioral performance

The proportion of correct responses in Exp. 2 is shown in **Figure 1D**. Importantly, the pattern of behavioral results replicated Exp. 1. Consistent with an influence of top-down control on distraction, there was a significant interaction between validity, load, and congruency [*F*(1,12) = 5.73, *p* = 0.034, $\eta_{partial}^{2}$ = 0.32]. Specifically, behavioral distraction was eliminated on valid cue trials: there were no significant main effects or interactions of flanker congruency when cues were valid (*p* > 0.08, $\eta_{partial}^{2}$ < 0.24). In addition, there was a significant interaction between flanker congruency and load when cues were invalid [*F*(1,12) = 8.60, *p* = 0.013, $\eta_{partial}^{2}$ = 0.42]. *Post hoc t*-tests revealed that this interaction on invalid trials was driven by a significant effect of flanker congruency under low load conditions [*M* = 0.05, SEM = 0.01, *t*(12) = 3.12, *p* = 0.009, *d* = 0.87]. In contrast, there was no effect of flanker congruency under high load conditions [*M* = -0.04, SEM = 0.03, *t*(12) = -1.58, *p* = 0.14, *d* = 0.44].

#### Event-related potentials

**FIGURE 5:**
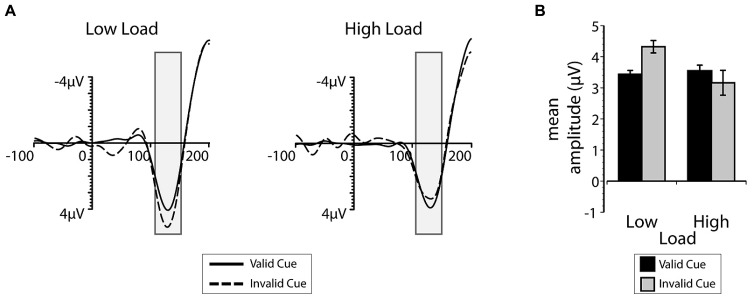
**(A)** The mean ERP-waveform over electrodes contralateral to the presentation of the irrelevant flanker between 100 ms prior to and 200 ms after the presentation of the search display. The gray bars indicate the average (45 ms) time window used to calculate the mean P1 amplitude. Valid trials are depicted with the solid line, invalid trials with the dashed line. **(B)** The mean P1 amplitude contralateral to the irrelevant flanker, plotted as a function of cue validity (valid in black and invalid in gray) and load. Error bars plotted within this figure represent the standard error of the mean appropriate for within-subjects comparisons (Loftus and Masson, 1994).

The group average ERPs and P1 mean amplitudes recorded at occipital and parietal-occipital electrodes contralateral to the position of the task-irrelevant flanker are plotted as a function of cue validity and search load in **Figure 5**. Visual inspection of both the ERP time-course and mean amplitudes suggest that there was an interaction between search load and top-down expectation during early sensory processing. When perceptual load was low, the P1 to the flanker was larger when the cue was invalid compared to when it was valid. When perceptual load was high, there was little or no difference in the P1 amplitude as a function of cue validity. There was also a significant interaction between validity and load on the P1 mean amplitudes [*F*(1,12) = 5.01, *p* = 0.045, $\eta_{partial}^{2}$ = 0.30]. *Post hoc t*-tests indicated that this interaction was driven by the smaller P1 amplitude on valid low load trials (*M* = 3.43 μV) compared to the invalid low load trials [*M* = 4.32 μV; *t*(12) = -4.25, *p* = 0.001, *d* = 1.17], but no influence of cue validity under high load [*t*(12) = 0.72, *p* = 0.49, *d* = 0.20]. Also consistent with the interpretation that accurate expectations eliminate behavioral distraction regardless of perceptual load, there was no difference between low and high load P1 amplitudes associated with the flanker on valid trials [*t*(12) = 0.35, *p* = 0.734, *d* = 0.09]. An additional analysis was also conducted on the mean amplitude of the N1 ERP component and it exhibited a qualitatively similar, but not statistically reliable, pattern. Taken together, the results from Exp. 2 support the interpretation that top-down expectation and perceptual processing demands interacted to influence sensory processing of the irrelevant flanker within the first 130 ms after the search display was presented.

#### Control analyses

Overall, the observed patterns of behavioral performance, fMRI responses in the dorsal attention network, and visual cortical responses measured with both fMRI and EEG are consistent with the idea that perceptual distraction is determined by the interaction between perceptual load and top-down expectations about task demands. The finding that predictive cues eliminated distraction under low load is inconsistent with a strict interpretation of the load theory assumption that the allocation of perceptual resources is automatic and exhaustive. However, load theory could potentially account for the interaction between perceptual difficulty and top-down expectation if it were assumed that the size of the resource pool is flexible and may change due to alertness and other factors (Lavie and Tsal, 1994). According to this modified load theory view, the predictive cues used here may result in the recruitment of a smaller resource pool when the cue indicates that the difficulty of the search display is likely to be low and a larger resource pool when the cue indicates that the likely difficulty will be high. Once the size of the resource pool is determined, the available resources are then distributed according to the automatic and exhaustive allocation scheme. This alternative account, which we refer to as the flexible resource pool hypothesis, makes a number of specific predictions about the effect of the cues on behavioral performance, cue-evoked BOLD responses in the dorsal attention network, and responses in visual cortex.

***Behavioral performance***. The flexible resource pool hypothesis makes four predictions about the effect of the cues on overall performance and an additional four predictions about the effect of the cues on the magnitude of flanker interference. First, if more resources are recruited when participants are given a high load cue, additional resources should be available to identify the target and overall accuracy should be higher on valid high load trials than invalid high load trials. However, there was no effect of cue validity on high load trials in Exp. 1A [*t*(13) = 0.28, *p* = 0.78, *d* = 0.07] or in Exp. 2 [*t*(12) = 0.22, *p* = 0.83, *d* = 0.06]. The lack of a difference in these conditions is unlikely due to floor effects because performance is well above chance. Second, if more resources are recruited when given a high load cue, additional resources should be available to identify the target and accuracy should be higher on valid high load trials compared to when no expectation is generated, as in the non-predictive high load trials. Contrary to this prediction, overall performance did not differ between the high load valid and high load non-predictive conditions [Exp. 1A versus Exp. 1B; *t*(48) = -0.37, *p* = 0.27, *d* = 0.12]. Similarly, there was no difference in overall performance between the invalid and non-predictive low load groups [Exp. 1A versus Exp. 1B; *t*(48) = 0.79, *p* = 0.43, *d* = 0.25]. Third, if fewer resources are recruited when given a low load cue, insufficient resources should be available to identify the target and accuracy should be lower on invalid high load trials compared to the non-predictive high load group. However, overall accuracy between invalid and non-predictive high load conditions was not different [Exp. 1A versus Exp. 1B; *t*(48) = -0.53, *p* = 0.29, *d* = 0.17]. Fourth, if fewer resources are available when expecting low load, fewer resources should be available to identify the target and accuracy should be lower on valid low load trials compared to the non-predictive low load group. However, there was no difference in performance between valid and non-predictive groups in the low load conditions [Exp. 1A versus Exp. 1B; *t*(48) = 1.10, *p* = 0.28, *d* = 0.35].

The predictions pertaining to flanker interference are as follows. First, if fewer resources are available when expecting low load than when expecting high load, there should be fewer excess resources to “spill-over” to irrelevant flankers on valid low load trials compared to invalid low load trials (i.e., low load display preceded by a high load cue). Consistent with this prediction, we observed less interference under valid low load conditions than invalid low load conditions in Exp. 1A [*F*(1,13) = 12.22, *p* = 0.004, $\eta_{partial}^{2}$ = 0.48] and Exp. 2 [*F*(1,12) = 6.27, *p* = 0.028, $\eta_{partial}^{2}$ = 0.34]. Second, more interference should be observed in valid high load conditions compared to invalid high load conditions because the high load cue would result in the recruitment of more resources and subsequently permit more excess resources to “spill over” to process the flanker. However, there was no significant congruency effect (i.e., distraction effect) in the valid high load condition in Exp. 1A [*t*(13) = 0.33, *p* = 0.746, *d* = 0.40] or Exp. 2 [*t*(12) = 1.04, *p* = 0.320, *d* = 0.35]. Similarly, an interaction between flanker congruency and cue validity under high load displays was not observed in Exp. 1A [*F*(1,13) = 0.25, *p* = 0.625, $\eta_{partial}^{2}$ = 0.02] or Exp. 2 [*F*(1,12) = 2.93, *p* = 0.113, $\eta_{partial}^{2}$ = 0.20]. Third, a smaller resource pool following a low load cue should result in a smaller amount of “spill-over” on valid low load trials compared to non-predictive low load conditions. Consistent with this interpretation, there was a significant difference between experiments Exp. 1A and Exp. 1B in flanker interference [*F*(1,48) = 8.62, *p* = 0.005, $\eta_{partial}^{2}$ = 0.15]. Direct comparisons revealed that this effect was driven by a difference in performance between congruent and incongruent flanker conditions in the non-predictive experiment [*M* = 0.05, SEM = 0.01, *t*(35) = 4.57, *p* < 0.001, *d* = 0.76], but not in the valid cue condition of Exp. 1A [*M* = 0.01, SEM = 0.02, *t*(13) = 0.58, *p* = 0.57, *d* = 0.15]. Fourth, the recruitment of extra resources in response to a high load cue predicts more flanker interference on invalid low load trials than on non-predictive low load trials. Contrary to this prediction, there was no difference in flanker interference between invalid low and non-predictive low load conditions [Exp 1A versus Exp 1B; *F*(1,48) = 2.60, *p* = 0.11, $\eta_{partial}^{2}$ = 0.05].

***Cue-evoked BOLD responses in the dorsal attention network***. The flexible resource pool hypothesis predicts that high load cues should recruit more resources, a recruitment likely mediated by attentional control systems. This predicts that high load cues should generally result in larger BOLD responses than low load cues. We tested this hypothesis by conducting a whole-brain analysis directly comparing high load cue-only trials versus low load cue-only trials. This contrast did not reveal any areas as being more active on high load trials than low load trials.

***BOLD and ERP responses in visual cortex***. The flexible resource pool hypothesis outlined above proposes that the pool of resources recruited should be proportional to the expectation of the task demand (e.g., expect high load = more resources recruited). Once a display is presented, resources are allocated to relevant information and any unused resources are automatically allocated to task-irrelevant information. This proposal makes four specific predictions about the patterns of BOLD activity in regions of visual cortex that selectively represent both relevant (MOG_rel > irrel_) and irrelevant locations (LinG_irrel > rel_). First, if expectations result in the differential recruitment of resources from a flexible resource pool, high and low load cues should evoke different BOLD responses in MOG_rel > irrel_ and LinG_irrel > rel_. Direct comparisons between the cue-types (low versus high) within each ROI in visual cortex revealed that the cues did not differentially modulate responses on cue-only trials within either MOG_rel > irrel_ [*t*(12) = 0.57, *p* = 0.58, *d* = 0.15] or LinG_irrel > rel_ [*t*(12) = 0.50, *p* = 0.63, *d* = 0.16]. Second, the flexible resource hypothesis predicts that activity in LinG_irrel > rel_ should be greater on invalid low load trials (cued high) than on valid low load trials because there should be more available resources to “spill over” to irrelevant information, but no difference was observed [*t*(12) = -0.75, *p* = 0.47, *d* = 0.21]. Third, BOLD responses in LinG_irrel > rel_ should be greater on valid high load trials than invalid high load trials, but no difference was observed [*t*(12) = 1.93, *p* = 0.076, *d* = 0.54]. Fourth, the flexible resource pool hypothesis predicts that there should be more excess resources available on invalid low load trials than on invalid high load trials and, as a result, activity in LinG_irrel > rel_ should be larger on invalid low load trials than invalid high load trials. However, a direct comparison revealed no significant difference in BOLD responses between low and high load on invalid trials [*t*(12) = 1.63, *p* = 0.13, *d* = 0.45].

The flexible resource pool hypothesis makes two predictions about the pattern of ERP responses. First, the flexible resource hypothesis predicts that there should be more resources available to “spill over” to the irrelevant flanker on invalid low load trials compared to valid low load trials. Consistent with this prediction, the P1 ERP component contralateral to the flanker was greater for invalid compared to valid low load trials [*t*(12) = 4.25, *p* = 0.001, *d* = 1.18]. Second, this alternative hypothesis also predicts fewer resources should be available to “spill over” to the irrelevant flanker on invalid high load trials compared to valid high load trials. Contrary to the flexible resource hypothesis, cue validity had no effect on the P1 ERP component contralateral to the flanker on high load trials [*t*(12) = 0.72, *p* = 0.49, *d* = 0.20].

***Summary of control analyses***. The flexible resource pool hypothesis proposes that predictive cues flexibly engage different amounts of resources that are automatically allocated based on the perceptual difficulty of the task-relevant display. This hypothesis makes 15 key predictions about behavior, visual evoked responses measured with fMRI BOLD and measured with ERP. Overall, only three of the 15 predictions were supported: (1) distraction was smaller when cues validly predicted low load compared to when cues were invalid, (2) distraction was smaller when cues validly predicted low load compared to the distraction observed in the non-predictive control group, and (3) the P1 associated with the irrelevant flanker was larger on invalid cue low load trials than on valid cue low load trials. Importantly, while these three significant tests are consistent with the flexible resource pool hypothesis of load theory, they are also consistent with the notion that the allocation of resources is mediated by the interaction between the perceptual demands of the task and one’s internal expectations. Specifically, the smaller amount of behavioral interference observed when the cues were valid compared to the behavioral interference observed when the cues were invalid or non-predictive supports the interpretation that accurate top-down expectation facilitates the filtering of task irrelevant information, even when perceptual difficulty is low. In addition, the larger sensory evoked P1 contralateral to the irrelevant flanker on invalid low load trials supports the notion that load can dictate distraction when participants’ expectations are violated. This violation results in greater distraction when perceptual load is low compared to when load was high.

Of course, given that many of the results did not support the flexible resource pool hypothesis, it is tempting to conclude that this hypothesis is not a viable explanation of the results reported here. However, because many of the tests were null results, one could justifiably argue that the flexible resource hypothesis was not supported because the experiments reported here were underpowered. If the lack of support for the flexible resource hypothesis was solely due to a lack of statistical power, then one could reasonably expect the effect sizes for the null results to be in the medium or large range (Cohen, 1992). However, the reported effect sizes generally do not support this claim. The 12 predictions of the flexible resource pool hypothesis that failed to receive support were tested using 18 separate hypothesis tests. Only one of these tests had an effect size considered to be large, four had medium effect sizes, and 13 had small effect sizes. Thus, while we cannot definitively rule out the flexible resource hypothesis as an account for the observed pattern of data, the proportion of predictions that were not confirmed, the distribution of effect sizes, and the fact that the predictions that were confirmed are also explained by an interaction between perceptual demand and top-down expectation support the notion that the flexible resource hypothesis is a less parsimonious explanation of the effect of top-down expectations on perceptual distraction.

## DISCUSSION

### ACCURATE EXPECTATIONS DIMINISH DISTRACTION

The experiments reported here tested load theory’s assertion that the perceptual processing of task-irrelevant information is determined by the automatic and exhaustive “spill over” of resources dictated by the perceptual capacity required to process task-relevant information. The primary result was that the extent to which task-irrelevant information was processed – measured by behavior, fMRI, and ERPs – was modulated by the interaction between the perceptual demands of the task and the accuracy of one’s explicit top-down expectations about the task’s difficulty. Behavioral distraction was driven by the perceptual demands of the task only when perceptual load was invalidly cued or when the cues were non-predictive. In contrast, when the cues were valid predictors of task demands, there was no evidence of distraction, even under low load conditions. Paralleling the behavioral data, correct advance knowledge of search difficulty eliminated any influence of perceptual load on fMRI responses in visual areas and on ERP responses measured at electrodes contralateral to the flanker location.

In contrast to load theory, the evidence reported here suggests that task-irrelevant information was effectively filtered out when expectations were accurate, even when the perceptual task demands were low. However, perceptual processing of relevant and irrelevant information was determined by the demands of the task-relevant information when top-down expectations were violated or otherwise unavailable.

An alternative interpretation of load theory proposes that the amount of resources available for perceptual processing is flexible and the quantity recruited on a given trial depends on expected task demands. According to this modified load theory view, the predictive cues used here may result in the recruitment of a smaller resource pool when the cue indicates that the difficulty of the search display is likely to be low and a larger resource pool when the cue indicates that the likely difficulty will be high. Once the size of the resource pool is determined, the available resources are then distributed according to the automatic and exhaustive allocation scheme. This flexible resource pool hypothesis makes 15 specific predictions about our data, but only three were found to be consistent with this alternative account. Importantly, these three predictions are also consistent with the notion that perceptual processing demands and top-down expectancies interact to determine the amount of distraction. Based on both the main findings and the control analyses, load theory does not seem to offer the most parsimonious explanation for the data. The cue-induced reduction in distraction in the low load condition also cannot be explained by a dilution of processing resources (e.g., Tsal and Benoni, 2010) because the search displays were the same on valid and invalid trials. Instead, the present results are more consistent with the notion that top-down biases, which can be induced by either competitive interactions (e.g., Torralbo and Beck, 2008; Scalf et al., 2013) or expectation (Johnson et al., 2002; Serences et al., 2004; Theeuwes et al., 2004; McNab and Klingberg, 2007), play a major role in mediating perceptual distraction. While the present results parallel studies showing that predictive spatial and feature cues affect early perceptual processing (e.g., Luck et al., 1997; Kastner et al., 1998; Hopfinger et al., 2000; Giesbrecht et al., 2006), the novel contribution of the present work is the demonstration that cues that engender explicit expectations about task difficulty on a trial-by-trial basis can interact with perceptual demands to influence the perceptual processing of task-irrelevant information. Specifically, our results demonstrate that accurate top-down expectations resulted in effective perceptual biasing in favor of relevant stimuli over irrelevant stimuli in both low and high load conditions, and that perceptual load determined distractor processing when explicit top-down expectations were violated or not available.

The finding that accurate trial-by-trial cue-generated expectations about task difficulty can attenuate perceptual distraction reported here stands in contrast to other studies in which search difficulty is blocked. In blocked tasks, subjects presumably have accurate expectations about task difficulty on all trials, yet perceptual distraction is typically observed (e.g., Lavie, 1995; Lavie and Cox, 1997; Lavie et al., 2004). There are two key aspects of our methods that may be responsible for this apparent discrepancy in the results: (1) the trial-by-trial cueing and (2) the instructions to actively use the information provided by the cue to perform the task. When search load is blocked, participants may be less motivated to actively use their knowledge about the difficulty of the task on a trial-by-trial basis. Instead, they may adopt a more passive mode of attention, allowing perceptual task demands to dictate the allocation of resources. In contrast, the trial-by-trial cueing and the instruction to actively use the information provided by the cue in the current experiment may have increased the engagement of top-down attentional control systems that, in turn, facilitated distractor filtering. While speculative, there is evidence consistent with this interpretation showing that expectations about the spatial location of the target generated by trial-by-trial cues and expectations about task demands generated by trial history can modulate the amount of behavioral distraction (e.g., Johnson et al., 2002; Theeuwes et al., 2004; Biggs and Gibson, 2010).

In addition to the intermixing of high and low load trials within the experimental blocks, another key methodological difference between the present experiments and many perceptual load studies reported in the literature is the choice of dependent measure. Typical perceptual load studies (e.g., Lavie, 1995; Lavie and Cox, 1997; Lavie et al., 2004), including those that have shown interactions between expectancies and the efficiency of selective attention (e.g., Johnson et al., 2002; Theeuwes et al., 2004), use response time (RT) as the dependent measure. In contrast, we used accuracy. The rationale for choosing accuracy as the dependent measure rather than RT was to use a measure sensitive to changes in perceptual processing while also minimizing speed-accuracy tradeoffs and motor biases that can occur when using reaction time as the dependent measure (e.g., Santee and Egeth, 1982; Prinzmetal et al., 2005; Bisley and Goldberg, 2006). The present work is not the first to use accuracy as a dependent measure to investigate perceptual load effects on distractor processing (Cartwright-Finch and Lavie, 2007; Konstantinou and Lavie, 2013), but it is nevertheless important to consider the implications that this choice has on the interpretation of our results. For instance, while we observed the elimination of the congruency effect on valid trials it is possible that accuracy is missing some aspect of flanker processing that can only be measured with RT (we thank Reviewer 1 for raising this possibility). Importantly, the key implication of our findings, namely that efficient selective attention depends on both accurate expectancies and perceptual demands, does not hinge on the complete elimination of the congruency effect. Indeed, while we cannot rule out the possibility that there is some residual flanker processing that could be detected with RT, even an attenuated congruency effect on valid low load trials would be inconsistent with a strong prediction of load theory. Moreover, we observed the typical reduction in the congruency effect under high load in conditions in which no expectations could be formed (Exp. 1B). Thus, while it may be the case that some aspects of our results are unique to the use of accuracy as a dependent measure, our results nevertheless demonstrate that accurate expectancies can mitigate some aspects of the interference caused by task-irrelevant information under low load conditions.

A final factor to consider when interpreting the results of the present experiments is the contribution of eye movements. Unfortunately, we did not have the capacity measure eye movements at the time Exp. 1 was conducted. Nevertheless, there are three reasons that rule out the confounding effects of eye movements. First, when a saccade is made, the movement alters the relative retinotopic position of the relevant and irrelevant spatial locations. If this happened frequently during the experiment, then we should not have been able to observe the greater perceptual selectivity (Relevant BOLD > Irrelevant BOLD) in visual cortical responses under high load compared to low load conditions in Exp. 1A. For example, if observers systematically moved their eyes more on high load trials, this would result in more blurred BOLD activations across stimulus locations and we might expect less selectivity under high load compared to low load. Second, if the incentive to move ones’ eyes was greater in one load condition compared to the other, one might expect differences in the high and low load BOLD responses in human frontal eye fields (FEF) and parietal cortex. However, this was not observed in the whole brain contrasts. Third, in Exp. 2 we were able to monitor eye movements to some extent using EOG and excluded trials in which eye movements and blinks occurred (see Materials and Methods). Importantly, Exp. 2 replicated the basic behavioral finding and showed modulation of the P1 ERP component.

### INVOLVEMENT OF THE DORSAL ATTENTION NETWORK

If top-down control mechanisms mediate the pattern of behavioral interference and visual cortical responses observed here, then regions of the dorsal attention network should also be modulated by the interaction between cue validity and task demands. Consistent with this line of reasoning, the dorsal attention network was engaged by both low and high load cues. While activation in this network did not show differential preparatory responses as a function of the expected difficulty, activity in this network was influenced by the interaction between perceptual demand and top-down expectation on trials that included a search display. Indeed, left IPS/SPL exhibited a larger BOLD response on invalidly cued low load trials compared to all other conditions. Importantly, this condition also corresponded to the only condition in which there was behavioral distraction, reduced perceptual selectivity measured with BOLD responses in visual cortex, and enhanced sensory processing of the irrelevant flankers measured with ERP. However, due to the low temporal resolution of fMRI, the interaction in dorsal frontoparietal cortex is consistent with several possible mechanisms: the top-down control over early perceptual selectivity, later post-perceptual distractor inhibition, or changes in the efficiency of sensory-motor response transformations (Gnadt and Anderson, 1988; Tunik et al., 2008; Martin et al., 2011). However, the pattern of IPS/SPL activation, the observed patterns of behavior and visual cortical responses measured with fMRI and ERP, and the established role of dorsal parietal cortex in the control and orienting of selective attention (Posner et al., 1984; Hopfinger et al., 2000; Corbetta and Shulman, 2002; Yantis et al., 2002; Friedman-Hill et al., 2003; Woldorff et al., 2004; Mevorach et al., 2006; Geng and Mangun, 2009; Akyürek et al., 2010; Melloni et al., 2012) support the conclusion that the cue led to the recruitment of dorsal parietal regions in support of filtering irrelevant information when expectations were valid. In cases when expectations were invalid, behavioral distraction was more likely on low load trials. The increased likelihood of distraction on invalid low load trials may have required a boosted response in dorsal parietal regions to filter irrelevant information. This role may come in the form of top-down biasing signals that influence visual processing (Bressler et al., 2008; Szczepanski et al., 2010), remapping of visual saliency (Geng and Mangun, 2009; Jerde and Curtis, 2013), or perceptual decision-making processes (Kayser et al., 2010; Liu and Pleskac, 2011; Hebart et al., 2012).

The interaction between perceptual demand and top-down expectation observed within dorsal frontal cortex (MFG/PreCs) revealed a larger response at later time points on invalidly cued high load trials compared to all other conditions. Given this region’s association with cognitive control (Koechlin et al., 2003), updating of the task-set (Sylvester et al., 2003; Derrfuss et al., 2004) and task difficulty (Demeter et al., 2011), the later sustained response could indicate the readjustment of the task set in order to cope with an unexpectedly more difficult search task.

### CORRELATIONS BETWEEN IPS RESPONSES AND INDIVIDUAL DIFFERENCES IN DISTRACTION

If IPS plays a role in providing top-down biasing signals to facilitate the filtering of task-irrelevant information, then when there is an increased likelihood for distraction, individuals with larger IPS/SPL responses should show less distraction. In the present study, the largest flanker interference was observed when a low-load search task was preceded by an invalid cue. We observed that those individuals who experienced the least distraction in this condition also exhibited larger BOLD responses in IPS/SPL compared to individuals who experienced the most distraction. In other words, individuals who more effectively recruited IPS/SPL after receiving incorrect information about the task were also more successful in filtering and resolving competition from irrelevant information. The negative correlation between behavioral interference and IPS/SPL responses is consistent with previous work demonstrating that activity in dorsal parietal cortex corresponds to conditions with greater attentional demand (Wojciulik and Kanwisher, 1999; Nobre et al., 2003) as well as work indicating that IPS is transiently involved when a shift in attention is required (Yantis et al., 2002). This finding is also consistent with proposals that IPS/SPL plays an important role in maintaining the focus of attention to filter irrelevant information when distraction is likely (Mevorach et al., 2006; Geng and Mangun, 2009; Melloni et al., 2012), and in facilitating efficient perceptual discrimination (Kayser et al., 2010; Liu and Pleskac, 2011; Hebart et al., 2012).

## CONCLUSION

We propose that the present findings are best explained by models of visual attention constrained by knowledge of the underlying neural mechanisms (e.g., Desimone and Duncan, 1995; Bundesen et al., 2005; Reynolds and Heeger, 2009). In these frameworks, the allocation of perceptual resources to task-relevant and task-irrelevant information is mediated by a combination of top-down biases that are based on one’s expectations and low-level competitive interactions (e.g., Torralbo and Beck, 2008; Kyllingsbæk et al., 2011; Parks et al., 2011; Nordfang et al., 2012; Scalf et al., 2013; Giesbrecht et al., 2014). Importantly, behavioral and neural responses to task-irrelevant information are not dictated by either expectations or low-level factors in isolation, but rather, determined by the dynamic interaction between these factors.

## Conflict of Interest Statement

The authors declare that the research was conducted in the absence of any commercial or financial relationships that could be construed as a potential conflict of interest.
